# Supplementary material for: Transformers and large language models are efficient feature extractors for electronic health record studies
Source: Commun Med (Lond). 2025 Mar 21;5:83. doi: 10.1038/s43856-025-00790-1 (PMC11928488; doi:10.1038/s43856-025-00790-1)
Supplement: Supplementary file 2 — Supplementary Information [file 43856_2025_790_MOESM2_ESM.pdf]

Transformers and large language models are efficient feature extractors for electronic health record studies: supplementary methods, figures, tables

Kevin Yuan*, Chang Ho Yoon*, Qingze Gu*, Henry Munby, A Sarah Walker, Tingting Zhu, David W Eyre

* *These authors contributed equally*

Supplementary Table S1: Glossary 2
Supplementary Methods 2
Comparator Classification by ICD-10 Codes 2
Free-Text Indication Pre-processing and Filtering 2
Traditional Classification Methods 3
BERT Classifier 3
Zero-Shot and finetuned LLM Classifier 3
Supplementary Note S1: Zero-Shot and Finetuned LLM Prediction Prompt (API) 4
Supplementary Note S2: Code and LUTs Repository 5
Supplementary Figure S1: Regex Rule Builder 6
Supplementary Figure S2: Labelling Coverage 7
Supplementary Figure S3: Training Set Size Effects 8
Supplementary Table S2: Prescribed Drugs 9
Supplementary Table S3: Prescribing Specialities 10
Supplementary Table S4: Patient Demographic 11
Supplementary Table S5: Uncommon and Common Indications 12
Supplementary Table S6: Inter-annotator Agreement Scores for Labelled Data 13
Supplementary Table S7: Category Distributions 14

Supplementary Table S1: Glossary

Definition of terms used throughout the manuscript:

**Category**	One of 11 different terms representing the infection source:<br><br>Urinary; Respiratory; Abdominal; Neurological; Skin and Soft Tissue; Ear, Nose and Throat (ENT); Orthopaedic; Other specific (i.e. another body site); Non-specific (i.e. no body site provided, e.g. "sepsis"), Prophylaxis, Not informative (i.e. text unrelated to the source of infection, e.g. "as instructed by Dr X").<br><br>A further, 12th category is included to reflect the presence/absence of any uncertainty in the original text string.
**(Antibiotic) Indication**	Free-text string recorded by the prescribing clinician alongside the drug, specifying the reason for prescribing the drug.
**Infection Source**	Source of infection or anatomical site affected (e.g. abdominal, urinary, etc), categorised into 11 groups as shown for "Category".
**Labels**	Manually verified categories for the free-text indications, serving as the ground-truth for our model training and testing.
**Multi-Label Classification**	Classification tasks where multiple outputs are possible. E. g. indication "chest/urinary" would fall into the categories "Respiratory" and "Urinary"
**Prescribing Uncertainty**	Uncertainty expressed by the clinician in the free-text indication field. Can be explicitly expressed by terms "unknown", "?" or similar; or implicit by specifying multiple unrelated infection sources e.g. "chest/urine".

Supplementary Methods

Comparator Classification by ICD-10 Codes

We first summarised all ICD-10 codes into broader concepts using the CCSR classification tool, grouping >70,000 ICD-10 codes into 544 categories. Two independent clinical researchers then developed a custom lookup table mapping CCSR categories to our 11 infection source groups (Supplement S3), with a third clinician resolving discrepancies.

Free-Text Indication Pre-processing and Filtering

A filter was applied to remove all non-sensical indications which could either be:

- Single character strings (e.g.. "a", "b")
- Just punctuation (e.g. ".", "…", "/", however the string "?" was kept)
- Just numbers (e. g. "1234")

All indications were then converted to lowercase to reduce the number of variants of the same string, as the prescribers often used inconsistent casing. No further preprocessing (like splitting) was applied.

Traditional Classification Methods

Regex Rules

The regex patterns for each category were built using the 50 most common indications for that infection source in the training data, with individually assigned error-rate thresholds for inexact matching and specified word boundaries for each string. This allowed for strict exact matching on abbreviations while permitting spelling mistakes for longer words. Word boundaries ensured that abbreviations were not matched when part of a longer word (e. g. avoiding finding UTI in roUTIne post-op, cUTIbacterium). Our tool, designed to automate the creation of complex regex queries based on a given reference set and user specifications, an example template can be found in *Supplementary S1*. The assisted regex builder simplified creating complex matching rules for infection sources, extracting the most common indications for each category and exporting them into a pre-populated table with parsing options for additional user input. Users can then modify these rules: adding word boundaries for precise matches, setting error rates (e.g., zero for abbreviations), and excluding redundant words. The edited table is then read back and converted into complex regex-matching strings for each category. This allows for medical experts to build and modify complex matching rules without needing to understand and debug error-prone regular expressions.

BERT Classifier

We evaluated the performance of the original generic "uncased base BERT" model, pre-trained on the BooksCorpus and English Wikipedia and a domain-specific "Bio+Clinical BERT", pre-trained on biomedical and clinical text sourced from PubMed, PubMed Central and MIMIC-III v1.4 notes[22,23]. Both pre-trained models were fetched from the HuggingFace model hub (uploaded on the 18-June-2019 and 28-February-2020), and finetuned using the HuggingFace "transformers" library with indications as input and the source categories as output [21].

Zero-Shot and finetuned LLM Classifier

We developed prompts for GPT4, comprised of instructions and the target categories, asking the model to complete the categories (specific prompt in ***Error! Reference source not found.****S2*)[24]. We made several iterations to the prompt on a subset of the training data, aiming to increase the model's understanding of the task. Given the model's generative nature, we accessed GPT4 through the API and supplied inputs to create more structured, deterministic and less creative answers. Specifying a rigid output format is crucial for a multi-label task. We therefore instructed the model to present its prediction output in JSON format, using the original indication as the key and the categories as a list of values. To prevent the model from creating new categories, we penalised it for returning new tokens not seen in the text (i.e. the prompt) by setting a higher `presence penalty'. A fixed `seed' and lower `temperature' were chosen to coerce the model into returning more deterministic and reproducible answers[32]. The same hyperparameters were used for prediction with the fine-tuned GPT3.5 model, aiming to increase determinism and reproducibility.

Supplementary Note S1: Zero-Shot and Finetuned LLM Prediction Prompt (API)

System prompt:

*You are a helpful and precise UK medical expert; you have been given a list of indications describing why antibiotics were prescribed to patients in a hospital. You have been asked to **label** these indications into categories.*

You can only ***choose*** *from these categories which are: Urinary, Respiratory, Abdominal, Neurological, Skin Soft Tissue, Ent, Orthopaedic, Other Specific, No Specific Source, Prophylaxis, Uncertainty, Not Informative*

Multiple categories are allowed.
When returning your answer, please return a json

User prompt:

This is the list of indications, return a json with the categories (multiple allowed) for each indication.

"abdo pathology",
"sepsis ?hap",
"artholin abscess",
[...]

The results are then parsed from the JSON-formatted response:

```
{
        "abdo pathology": ["Abdominal"],
        "sepsis ?hap": ["Respiratory", "Uncertainty"],
        "artholin abscess": ["Skin Soft Tissue"],
        [...]
}
```

Notes:

Both models occasionally returned additional categories, outside of the 11 infection categories + uncertainty. The prompt has been designed to enforce the model to only return categories specified in the input. Categories returned outside of the 12 specified were ignored.

Supplementary Note S2: Code and LUTs Repository

ICD-10 to Infection Source Mapping Table

https://github.com/kevihiiin/EHR-Indication-Processing/blob/main/00_Data/LUTs/icd10_ccsr_mapping.csv

Or on Zenodo:

https://doi.org/10.5281/zenodo.13987740

Supplementary Figure S1: Regex Rule Builder

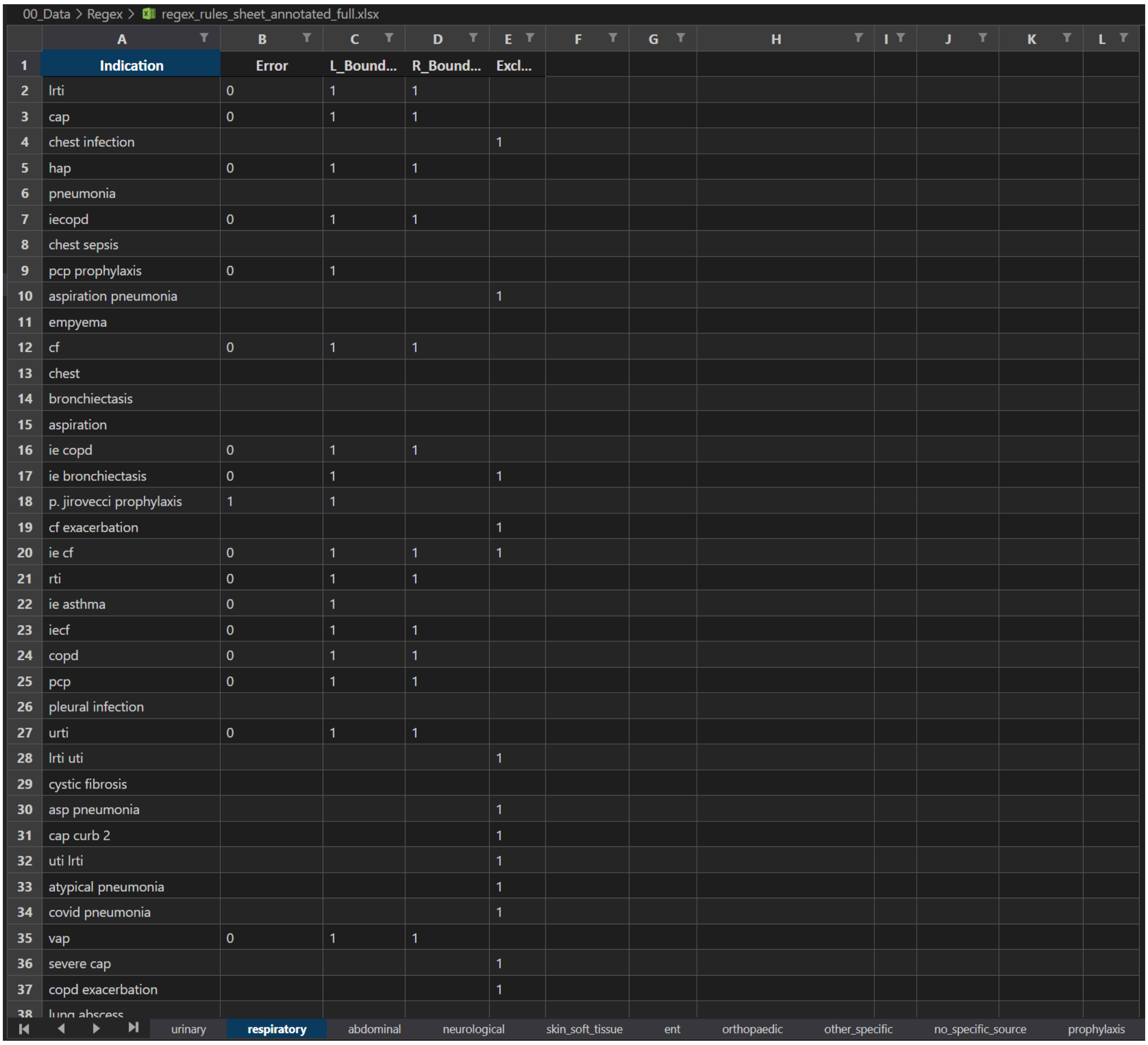
00_Data > Regex > regex_rules_sheet_annotated_full.xlsx

	A	B	C	D	E	F	G	H	I	J	K	L
1	Indication	Error	L_Bound...	R_Bound...	Excl...							
2	lrti	0	1	1								
3	cap	0	1	1								
4	chest infection				1							
5	hap	0	1	1								
6	pneumonia											
7	iecopd	0	1	1								
8	chest sepsis											
9	pcp prophylaxis	0	1									
10	aspiration pneumonia				1							
11	empyema											
12	cf	0	1	1								
13	chest											
14	bronchiectasis											
15	aspiration											
16	ie copd	0	1	1								
17	ie bronchiectasis	0	1		1							
18	p. jirovecci prophylaxis	1	1									
19	cf exacerbation				1							
20	ie cf	0	1	1	1							
21	rti	0	1	1								
22	ie asthma	0	1									
23	iecf	0	1	1								
24	copd	0	1	1								
25	pcp	0	1	1								
26	pleural infection											
27	urti	0	1	1								
28	lrti uti				1							
29	cystic fibrosis											
30	asp pneumonia				1							
31	cap curb 2				1							
32	uti lrti				1							
33	atypical pneumonia				1							
34	covid pneumonia				1							
35	vap	0	1	1								
36	severe cap				1							
37	copd exacerbation				1							
38	lung abscess											

urinary | respiratory | abdominal | neurological | skin_soft_tissue | ent | orthopaedic | other_specific | no_specific_source | prophylaxis

Figure S1: Regex Rule Builder example user specification (for respiratory infection). *The columns allow the user to specify a non-default error rate for abbreviations, set left and right word boundaries and exclude duplicated words. One sheet per category. The default allowed error rate (for any insertion, deletion or character substitutions) was 10% with a maximum of 2 and minimum of 1 per string. Left and right boundaries require the string to exist as a separate word (after separating words using spaces, commas, hyphens and slashes). The exclusion column allows words already captured by other strings to be ignored, e.g. "chest infection" as "chest" is already present.*

An **example annotated rule sheet** can be found on Github and Zenodo:
https://github.com/kevihiiin/EHR-Indication-Processing/blob/main/02_Models/01_Baseline/Regex/Regex_rules_sheet_annotated.xlsx

https://doi.org/10.5281/zenodo.13987740

Supplementary Figure S2: Labelling Coverage

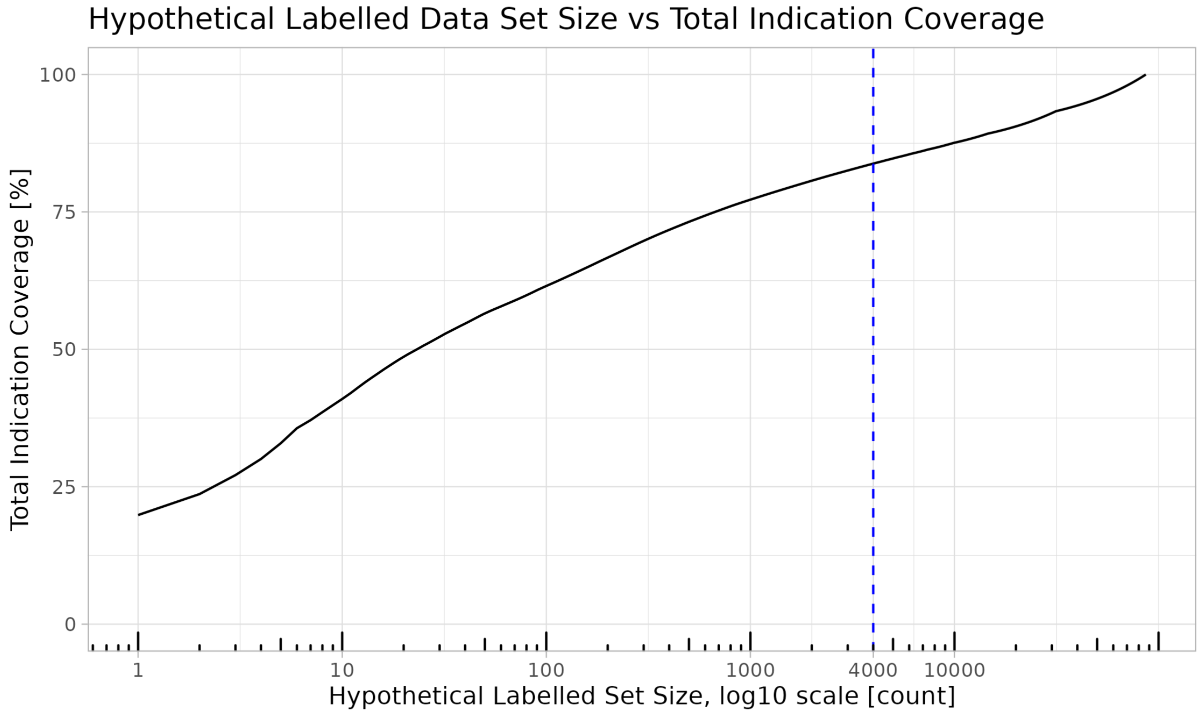

Figure S2: Hypothetical total coverage of data given a set of labelled data*. Labelling the most common 4000 text strings would cover 84% of the entire data set. If we were to label the most common 10 000 strings, we would achieve a coverage of 90%.*

Supplementary Figure S3: Training Set Size Effects

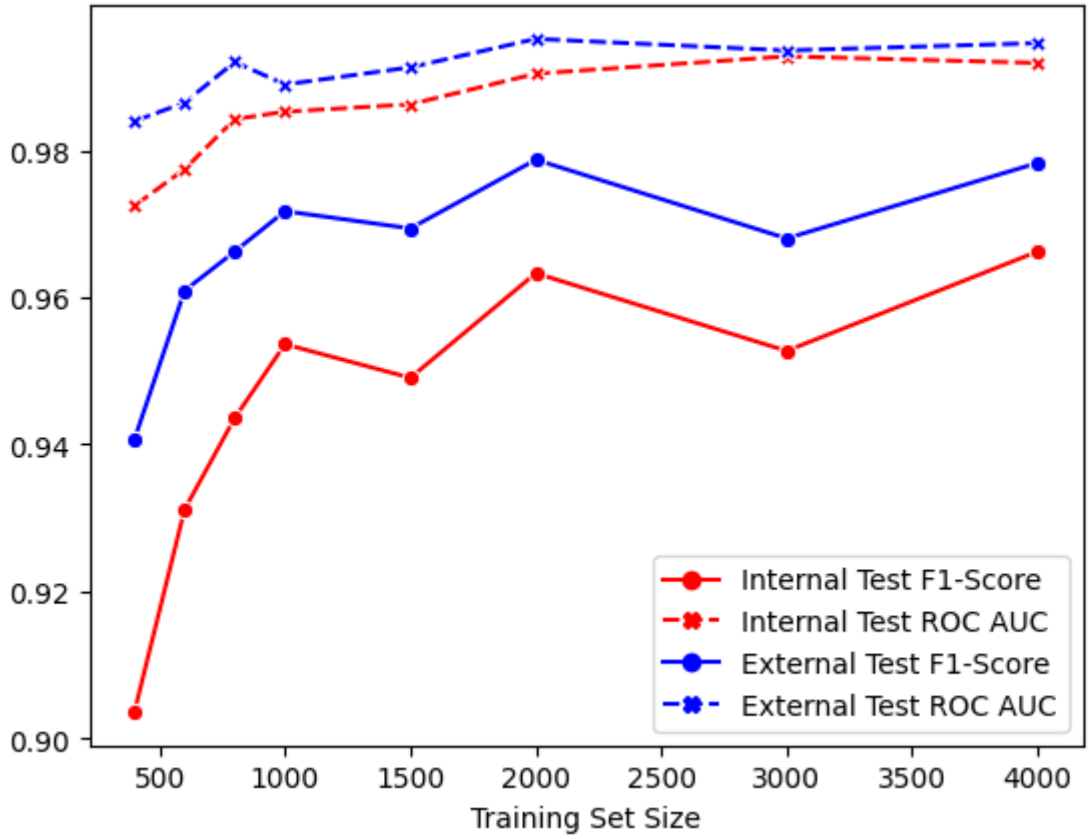

Figure S3: Training set size effect on the performance of Bio+Clinical BERT, evaluated on both F1-Score and ROC AUC*. The training was run on randomly sampled subsets of the training dataset of size [500, 1000, 1500, 2000, 3000, 4000] and evaluated on the same internal and external test sets (2000 samples each).*

Supplementary Table S2: Prescribed Drugs

Table S2: Ten most commonly prescribed drugs.

Drug [Oxford]	Number	Percentage
Co-amoxiclav	269945	33%
Gentamicin	70002	8%
Metronidazole	65094	8%
Ceftriaxone	58763	7%
Flucloxacillin	47537	6%
Amoxicillin	32151	4%
Ciprofloxacin	25145	3%
Vancomycin	23250	3%
Piperacillin + Tazobactam (Tazocin equivalent)	21666	3%
Clarithromycin	18607	2%

Drug [Banbury]	Number	Percentage
Co-amoxiclav	39114	35%
Ceftriaxone	11369	10%
Gentamicin	10251	9%
Amoxicillin	7297	7%
Clarithromycin	6525	6%
Flucloxacillin	5630	5%
Metronidazole	4851	4%
Doxycycline	4416	4%
Nitrofurantoin	3204	3%
Piperacillin + Tazobactam (Tazocin equivalent)	2862	3%

Supplementary Table S3: Prescribing Specialities

Table S3: Specialties of the prescribing clinicians, ordered to show the ten most active specialties.

Clinician Main Specialty [Oxford]	Number	Percentage
General Surgery	146719	18%
Acute General Medicine	98687	12%
Trauma and Orthopaedics	90719	11%
Acute Geratology	72371	9%
Clinical Haematology	46928	6%
Obstetrics	36431	4%
Neurosurgery	34666	4%
Infectious Diseases	30584	4%
Plastic Surgery	29334	4%
Urology	29258	4%

Clinician Main Specialty [Banbury]	Number	Percentage
Acute General Medicine	41312	37%
Acute Geratology	14912	13%
Gastroenterology	9128	8%
Cardiology	8682	8%
Infectious Diseases	7870	7%
Trauma and Orthopaedics	7642	7%
General Surgery	6803	6%
Emergency Medicine	4924	4%
Urology	2885	3%
Gynaecology	2287	2%

Supplementary Table S4: Patient Demographic

***Table S4: Patient** characteristics (Age, Sex, Ethnicity) across the Oxford and Banbury site. Values reported as median with inter quartile range intervals or percentages if applicable.*

**Characteristic**	**Oxford**, N = 171,460[1]	**Banbury**, N = 25,924[1]
**Age**	57 (36, 74)	67 (47, 81)
**Sex**		
F	94,721 (55%)	13,853 (53%)
M	76,738 (45%)	12,071 (47%)
**Ethnicity**		
Asian	4,525 (2.6%)	402 (1.6%)
Black	1,961 (1.1%)	139 (0.5%)
Mixed	1,315 (0.8%)	143 (0.6%)
Other	1,956 (1.1%)	149 (0.6%)
Unknown	44,155 (26%)	4,427 (17%)
White	117,548 (69%)	20,664 (80%)

[1]Median (IQR); n (%)

Supplementary Table S5: Uncommon and Common Indications

Table S5.1: Example list of uncommon indications. *Randomly sampled from all indications occurring more than 5 and less than 100 times. Occurrences less than 10 are truncated to <10 for statistical disclosure control.*

Uncommon Indication	Occurrence	Percentage
septic left knee	<10	<1%
abdo contamination	<10	<1%
infected femur	10	<1%
flu prophylaxis	63	<1%
forearm cellulitis	<10	<1%
mesenteric panniculitis	<10	<1%
intra-abdo inf	14	<1%
?dental infection	14	<1%
cap curb1	41	<1%
septic unknown source	<10	<1%

Table S5.2: Example list of common indications. *Showing the 10 most common indications.*

Common Indication	Occurrence	Percentage
perioperative prophylaxis	164058	20%
uti	31564	5%
lrti	28548	5%
sepsis	24192	4%
cap	23702	4%
prophylaxis	22602	4%
cellulitis	12115	2%
infection	12017	2%
intra-partum prophylaxis	10476	2%
chest infection	9331	2%

Supplementary Table S6: Inter-annotator Agreement Scores for Labelled Data

Table S6: Cohen's Kappa score for inter-annotator agreement for the labelled data. Calculated per category between the first and second clinical researcher, the last column being an average across the Cohen's Kappa score of all 12 classes.

	**Urinary**	**Respiratory**	**Abdominal**	**Neurological**	**Skin & Soft Tissue**	**ENT**	**Orthopaedic**	**Other Specific**	**No Specific Source**	**Prophylaxis**	**Uncertainty**	**Not Informative**	**Average**
Training Oxford	0.90	0.88	0.83	0.91	0.84	0.59	0.74	0.55	0.76	0.81	0.96	0.80	0.80
Testing Oxford	0.95	0.93	0.88	0.81	0.84	0.73	0.75	0.43	0.86	0.93	0.80	0.81	0.81
Testing Banbury	0.96	0.98	0.85	0.92	0.95	0.66	0.94	0.45	0.93	0.94	0.93	0.90	0.87

Supplementary Table S7: Category Distributions

Table S7: Distributions of the classes within the different data sets.

	Urinary	Respiratory	Abdominal	Neurological	Skin & Soft Tissue	ENT	Orthopaedic	Other Specific	No Specific Source	Prophylaxis	Not Informative
Training	6.26% (n=54882)	12.91% (n=113211)	6.33% (n=55486)	0.76% (n=6641)	4.43% (n=38843)	1.47% (n=12902)	1.66% (n=14522)	2.30% (n=20177)	35.40% (n=310479)	27.50% (n=241201)	0.98% (n=8598)
Oxford Test	5.79% (n=160)	13.53% (n=374)	8.64% (n=239)	0.83% (n=23)	4.74% (n=131)	1.84% (n=51)	1.99% (n=55)	3.18% (n=88)	32.77% (n=906)	25.46% (n=704)	1.23% (n=34)
Banbury Test	12.51% (n=305)	28.95% (n=706)	4.84% (n=118)	0.74% (n=18)	7.59% (n=185)	0.57% (n=14)	0.98% (n=24)	1.03% (n=25)	26.65% (n=650)	14.72% (n=359)	1.44% (n=35)

Supplementary Table S8: Per Category Prediction Scores

Table S8.1: Per category prediction scores on the internal Oxford test set. Values are reported in F1 score, Precision Recall AUC and ROC AUC where applicable.

Model	Metric	Urinary	Respiratory	Abdominal	Neurological	Skin & Soft Tissue	ENT	Orthopaedic	Other Specific	No Specific Source	Prophylaxis	Uncertainty	Not Informative
Regex	F1	0.87	0.63	0.63	0.68	0.76	0.54	0.3	0.11	0.78	0.93	0.20	0.00
XGBoost	F1	0.64	0.81	0.9	0.76	0.83	0.83	0.7	0.71	0.91	0.96	0.66	0.9
Base_BERT	F1	0.97	0.98	0.94	0.23	0.94	0.27	0.59	0.63	0.98	0.98	0.95	0.67
Bio_ClinicalBERT	F1	0.98	0.98	0.96	0.88	0.93	0.91	0.93	0.84	0.98	0.98	0.96	0.85
Fine-Tuned GPT3.5	F1	0.98	0.97	0.95	0.93	0.83	0.8	0.95	0.77	0.97	0.97	0.92	0.99
Zero-Shot GPT4	F1	0.98	0.96	0.83	0.88	0.87	0.79	0.87	0.30	0.34	0.94	0.78	0.30
Regex	PR	-	-	-	-	-	-	-	-	-	-	-	-
XGBoost	PR	0.62	0.87	0.91	0.68	0.89	0.82	0.65	0.72	0.97	0.99	0.69	0.83
Base_BERT	PR	0.98	0.99	0.97	0.69	0.95	0.85	0.94	0.77	0.99	0.99	0.97	0.92
Bio_ClinicalBERT	PR	0.99	1.00	0.97	0.91	0.98	0.93	0.96	0.88	0.99	1.00	0.99	0.94
Fine-Tuned GPT3.5	PR	-	-	-	-	-	-	-	-	-	-	-	-
Zero-Shot GPT4	PR	-	-	-	-	-	-	-	-	-	-	-	-
Regex	ROC	-	-	-	-	-	-	-	-	-	-	-	-
XGBoost	ROC	0.9	0.96	0.97	0.92	0.98	0.96	0.91	0.92	0.97	0.99	0.87	0.94
Base_BERT	ROC	0.98	1.00	0.98	0.97	0.99	0.96	0.99	0.91	0.99	0.99	1.00	0.98
Bio_ClinicalBERT	ROC	0.99	1.00	0.98	1.00	0.99	0.98	0.99	0.96	0.99	1.00	1.00	0.99
Fine-Tuned GPT3.5	ROC	-	-	-	-	-	-	-	-	-	-	-	-
Zero-Shot GPT4	ROC	-	-	-	-	-	-	-	-	-	-	-	-
Regex	Accuracy	0.98	0.79	0.89	0.99	0.97	0.96	0.89	0.36	0.76	0.96	0.32	0.97
XGBoost	Accuracy	0.96	0.94	0.98	1.00	0.98	0.99	0.99	0.98	0.92	0.97	0.95	1.00
Base_BERT	Accuracy	0.99	0.99	0.99	0.99	0.99	0.98	0.98	0.97	0.98	0.98	0.99	0.99
Bio_ClinicalBERT	Accuracy	1.00	0.99	0.99	1.00	0.99	1.00	1.00	0.99	0.98	0.98	0.99	1.00
Fine-Tuned GPT3.5	Accuracy	1.00	0.99	0.99	1.00	0.97	0.99	1.00	0.98	0.97	0.98	0.99	1.00
Zero-Shot GPT4	Accuracy	1.00	0.98	0.96	1.00	0.98	0.99	0.99	0.94	0.64	0.96	0.97	0.96

Table S8.2: Per category prediction scores on the external Banbury test set. Values are reported in F1 score, Precision Recall AUC and ROC AUC where applicable.

Model	Metric	Urinary	Respiratory	Abdominal	Neurological	Skin & Soft Tissue	ENT	Orthopaedic	Other Specific	No Specific Source	Prophylaxis	Uncertainty	Not Informative
Regex	F1	0.96	0.73	0.35	0.78	0.90	0.30	0.25	0.03	0.81	0.95	0.30	0.00
XGBoost	F1	0.67	0.8	0.86	1.00	0.91	0.87	0.76	0.63	0.92	0.98	0.72	0.87
Base_BERT	F1	0.99	0.99	0.92	0.76	0.96	0.73	0.63	0.65	0.98	0.98	0.99	0.79
Bio_ClinicalBERT	F1	0.99	0.99	0.95	1.00	0.96	0.92	0.91	0.92	0.99	0.97	0.99	0.87
Fine-Tuned GPT3.5	F1	0.98	0.98	0.95	1.00	0.91	0.81	0.90	0.70	0.98	0.96	0.95	1.00
Zero-Shot GPT4	F1	0.99	1.00	0.85	1.00	0.96	0.88	0.91	0.25	0.6	0.96	0.88	0.59
Regex	PR	-	-	-	-	-	-	-	-	-	-	-	-
XGBoost	PR	0.67	0.87	0.83	1.00	0.91	0.91	0.77	0.57	0.95	0.98	0.73	0.83
Base_BERT	PR	1.00	1.00	0.96	1.00	0.98	0.9	0.88	0.75	0.98	0.99	0.99	0.92
Bio_ClinicalBERT	PR	1.00	1.00	0.95	1.00	0.98	1.00	0.96	0.87	0.98	0.98	0.99	0.94
Fine-Tuned GPT3.5	PR	-	-	-	-	-	-	-	-	-	-	-	-
Zero-Shot GPT4	PR	-	-	-	-	-	-	-	-	-	-	-	-
Regex	ROC	-	-	-	-	-	-	-	-	-	-	-	-
XGBoost	ROC	0.86	0.93	0.94	1.00	0.96	0.96	0.90	0.93	0.97	0.99	0.87	0.94
Base_BERT	ROC	1.00	1.00	0.98	1.00	0.99	0.99	1.00	0.95	1.00	0.99	1.00	0.98
Bio_ClinicalBERT	ROC	1.00	1.00	0.98	1.00	0.99	1.00	0.98	0.97	0.99	0.99	1.00	0.99
Fine-Tuned GPT3.5	ROC	-	-	-	-	-	-	-	-	-	-	-	-
Zero-Shot GPT4	ROC	-	-	-	-	-	-	-	-	-	-	-	-
Regex	Accuracy	0.99	0.75	0.84	0.99	0.98	0.97	0.95	0.41	0.85	0.98	0.41	0.97
XGBoost	Accuracy	0.92	0.88	0.98	1.00	0.98	1.00	0.99	0.99	0.95	0.99	0.94	1.00
Base_BERT	Accuracy	1.00	0.99	0.99	1.00	0.99	1.00	0.99	0.99	0.99	0.99	1.00	0.99
Bio_ClinicalBERT	Accuracy	1.00	0.99	0.99	1.00	0.99	1.00	1.00	1.00	0.99	0.99	1.00	1.00
Fine-Tuned GPT3.5	Accuracy	0.99	0.99	0.99	1.00	0.98	1.00	1.00	0.99	0.99	0.99	0.99	1.00
Zero-Shot GPT4	Accuracy	1.00	0.98	0.96	1.00	0.98	0.99	0.99	0.94	0.64	0.96	0.97	0.96

Table S8.3: Averaged performance scores using weighted (reported in the main section) micro and macro averaging on the internal and external test set.

Model	Metric	Avg. Weighted (Oxford)	Avg. Micro (Oxford)	Avg. Macro (Oxford)	Avg. Weighted (Banbury)	Avg. Micro (Banbury)	Avg. Macro (Banbury)
Regex	F1-Score	0.71	0.55	0.54	0.74	0.57	0.53
Regex	ROC AUC	0.83	0.85	0.81	0.85	0.88	0.82
Regex	PR AUC	0.60	0.37	0.42	0.63	0.39	0.44
Regex	Accuracy	0.82	0.82	0.82	0.82	0.82	0.82
Regex	Total Accuracy	0.14	0.14	0.14	0.24	0.24	0.24
XGBoost	F1-Score	0.86	0.87	0.80	0.84	0.85	0.83
XGBoost	ROC AUC	0.96	0.97	0.94	0.94	0.96	0.93
XGBoost	PR AUC	0.90	0.91	0.80	0.87	0.87	0.83
XGBoost	Accuracy	0.95	0.95	0.95	0.94	0.94	0.94
XGBoost	Total Accuracy	0.72	0.72	0.72	0.68	0.68	0.68
Base_BERT	F1-Score	0.93	0.94	0.76	0.97	0.97	0.86
Base_BERT	ROC AUC	0.99	0.99	0.98	0.99	1.00	0.99
Base_BERT	PR AUC	0.97	0.97	0.92	0.98	0.98	0.94
Base_BERT	Accuracy	0.98	0.98	0.98	0.99	0.99	0.99
Base_BERT	Total Accuracy	0.88	0.88	0.88	0.95	0.95	0.95
Bio_ClinicalBERT	F1-Score	0.97	0.97	0.93	0.98	0.98	0.96
Bio_ClinicalBERT	ROC AUC	0.99	0.99	0.99	0.99	1.00	0.99
Bio_ClinicalBERT	PR AUC	0.98	0.98	0.96	0.98	0.98	0.97
Bio_ClinicalBERT	Accuracy	0.99	0.99	0.99	0.99	0.99	0.99
Bio_ClinicalBERT	Total Accuracy	0.94	0.94	0.94	0.97	0.97	0.97
GPT3.5	F1-Score	0.95	0.95	0.92	0.97	0.97	0.93
GPT3.5	ROC AUC	0.96	0.97	0.94	0.98	0.98	0.95
GPT3.5	PR AUC	0.91	0.91	0.86	0.94	0.94	0.88
GPT3.5	Accuracy	0.98	0.98	0.98	0.99	0.99	0.99
