# Supplementary material for: Transformers and large language models are efficient feature extractors for electronic health record studies
Source: Commun Med (Lond). 2025 Mar 21;5:83. doi: 10.1038/s43856-025-00790-1 (PMC11928488; doi:10.1038/s43856-025-00790-1)
Supplement: Supplementary file 3 — Description of Additional Supplementary File [file 43856_2025_790_MOESM3_ESM.pdf]

Description of additional supplementary files

File name: Supplementary Data 1
Description: Numerical results underlying the panels

File name: Supplementary Data 2
Description: Numerical results underlying the flow chart

File name: Supplementary Data 3

Description: True and predicted labels for both internal and external test sets. Raw values used to compute the metrics shown in the figure.

File name: Supplementary Data 4

Description: Numerical results underlying the Sankey diagram
